# Supplementary material for: Developing and Validating an Individual-Level Deprivation Index for Children’s Health in France
Source: Int J Environ Res Public Health. 2022 Dec 16;19(24):16949. doi: 10.3390/ijerph192416949 (PMC9816939; doi:10.3390/ijerph192416949)
Supplement: Supplementary file 1 [file ijerph-19-16949-s001.zip › Figure S1.pdf]

RESSOURCES EN CAS DE VULNÉRABILITÉ SOCIALE ET SANITAIRE EN PÉDIATRIE

Pourquoi poser ces questions pendant un soin ?
Où orienter en cas de besoin ?

LE SUIVI MÉDICAL
Des visites médicales systématiques permettent des actions de dépistage et de prévention tout au long de la vie. L'utilisation d'un carnet de santé est recommandée. Il peut être obtenu auprès du Conseil Départemental. Un enfant doit consulter un médecin avant 8 jours de vie, une fois par mois jusqu'à 6 mois, à 9 mois, 12 mois, 13 mois, 18 mois, à 2 ans, deux fois par an jusqu'à 6 ans, puis une fois par an jusqu'à l'âge adulte.
Ressources : Médecin Traitant, service de Protection Materno-Infantile du Conseil Départemental, Maison de Santé, Permanence d'Accès Aux Soins de Santé.

LA COUVERTURE SOCIALE
Il est important d'adapter ses prescriptions au risque qu'un patient ne puisse les recevoir. Certains soins sont mal remboursés par les assurances santé (dentaire, lunetterie, crèmes, collyres...) et nécessitent certaines précautions. L'entretien avec une Assistante Sociale peut informer sur les droits, éviter des facturations au patient et permettre la continuité des soins et des thérapeutiques.
Ressources : Caisse Primaire d'Assurance Maladie, Permanence d'Accès Aux soins de Santé.

LA SCOLARISATION DES ENFANTS DE 3 À 15 ANS
La scolarisation est obligatoire en France de 3 à 15 ans. Elle favorise l'apprentissage du français, l'intégration sociale et ensuite professionnelle.
Ressources : Mairie, École, Associations.

LE LOGEMENT
Un logement stable est nécessaire à l'organisation de la vie de famille, à la scolarisation et au parcours de santé.

Ressources : 115, Services Intégré d'Accueil et d'Orientation, Assistante Sociale du Conseil Départemental dans les Maisons Départementales de la Solidarité, à l'hôpital, Associations.
Outre l'inconfort, différents désordres dans le logement peuvent causer des maladies et accidents (asthme, traumatismes, brûlures, intoxication au plomb, au monoxyde de carbone...). Pour demander une réhabilitation du logement ou un relogement social, différentes procédures sont accessibles après une évaluation sociale ou environnementale.
Ressources : Le Pôle Départemental de Lutte contre l'Habitat Indigne, Médecin Traitant, Assistante Sociale du Conseil Départemental dans les Maisons Départementales de la Solidarité, Permanence d'Accès Aux soins de Santé Pédiatrique ou Mère-Enfant, Associations.

L'ACCÈS À L'ALIMENTATION
L'insécurité alimentaire est présente en France (> 10 % de la population). L'accès à la nourriture et à l'eau est un préalable à la majorité des projets de soins.
Ressources : Associations, Assistante Sociale du Conseil Départemental dans les Maisons Départementales de la Solidarité.

L'ENTOURAGE
L'assistance par l'entourage est importante pour s'occuper des enfants et l'organisation de certains soins (les déplacements, les démarches administratives, le soutien à la parentalité, le suivi médical...).
Ressources : Technicienne de l'Intervention Sociale et Familiale, Assistante Sociale du Conseil Départemental dans les Maisons Départementales de la Solidarité, à l'hôpital, Centre Sociaux, Associations de quartier.

Outil **ENVU**
Questionnnaire de repérage de la vulnérabilité sociale et sanitaire en pédiatrie

Guide à destination des soignants

**Les inégalités sociales affectent la santé dès le plus jeune âge.
Pour les réduire, il faut d'abord les repérer.
Transformer votre prescription en un soin, c'est l'adapter aux ressources de l'enfant et sa famille.**

Outil de dépistage des **EN**fants vivant en contexte socialement **VU**lnérable

Enfant : .... Date : ....

	ITEMS	RÉPONSES			
A.	Avez-vous un carnet de santé ou document de vaccination pour chacun de vos enfants (ici ou à la maison) ?	OUI: 0		NON: 8	
B.	Vos enfants sont-ils tous suivis (au moins une fois par an) par un médecin traitant (de famille, en PMI) ?	OUI: 0		NON: 14	
C.	Avez-vous une couverture maladie complète ? pour chacun de vos enfants?<br>PUMA : Protection Universelle Maladie (ancienne «sécurité sociale de base» et «CMU base»)<br>CSS : Complémentaire Santé solidaire (ancienne «ACS» et «CMU complémentaire»)<br>AME : Aide Médicale d'Etat	OUI complète (PUMA+mutuelle: **0**<br>OUI complète (PUMA+CSS): **4**<br>OUI (AME): **8**		NON aucune (même si démarches en cours ou assurance étrangère): **26**<br>NON incomplète (PUMA seule sans mutuelle ni CSS): **17**	
D.	Tous vos enfants, entre 3 et 15 ans, vont- ils à l'école ?	OUI: **0** (ou non concerné)		NON: **7**	
E.	Avez-vous un logement personnel ou familial (maison, appartement, chambre) ?	LOGEMENT STABLE: **0**	LOGEMENT INSTABLE (hébergement social ou dans l'entourage, squat): **17**	AUCUN LOGEMENT: **26**	
F.	Si logement stable, est-il en mauvais état, dégradé, dangereux (peintures écaillées, moisissures, risque d'électrisation, de traumatisme)?	NON: 0		OUI : 17	
G.	Connaissez-vous un travailleur social qui vous assiste (aide) dans vos démarches (administratives, sociales) ?			OUI: 6	
	Si non, en avez-vous besoin d'un ?	NON: 0		OUI: 9	
H.	Vivez-vous seul avec vos enfants (parent isolé) ?	NON: 0		OUI: 5	
I.	Vous arrive-t-il parfois de ne pas avoir assez à manger ?	NON: 0		OUI: 8	
J.	Y-a-t-il dans votre entourage des personnes de confiance sur qui vous pouvez compter pour vous aider (pour les déplacements, garder les enfants, permettre d'aller à des RDV, aider dans des démarches) ?	OUI: 0		NON: 9	
K.	Avez-vous changé de région (ou pays) au cours de deux dernières années ?	NON: 0		OUI: 2	
L.	[Évaluation par le soignant]<br>Les questions, explications au patient sont-elles limitées en français ?	NON: 0		OUI: 2	
				**TOTAL**	

TOTAL < 6

—

Pas de suspicion de fragilité sociale

susceptible de menacer l'accès aux soins et la santé

> Pas d'orientation, sauf demande spécifique (voir au dos)

TOTAL DE 6 À 25

—

Fragilité sociale manifeste

susceptible de menacer l'accès aux soins et la santé

> Proposer une orientation adaptée aux difficultés (voir au dos)

> En l'absence de suivi social, une évaluation et un accompagnement social peuvent être proposés.

TOTAL ≥ 26

—

Fragilité sociale importante

susceptible de menacer fortement l'accès aux soins et la santé

> Proposer une orientation spécialisée (ex. PASS pédiatrique ou mère-enfant)
